# Co-infection of *Cystoisospora suis* with enterotoxigenic *Escherichia coli* synergistically increases pathogenicity in weaned piglets

**DOI:** 10.1186/s13071-026-07291-7

**Published:** 2026-03-05

**Authors:** Zi-Ying He, Lian-Xiang Wang, Xiao-Ling Deng, Jia-Jia Tan, Ao Wang, Yi-Juan Huang, Hai-Yue Wu, Jun-Yuan Du, Dong-Fang Zhao, Rui-Qing Lin

**Affiliations:** 1 South China Agricultural University, Guangzhou, 510642 Guangdong People’s Republic of China; 2 Wen’s Group Academy, Wen’s Foodstuffs Group Co., Ltd., Guangdong Provincial Enterprise Key Laboratory of Healthy Animal Husbandry and Environment Control, Xinxing, 527400 Guangdong People’s Republic of China; 3 Foshan Standard Bio-Tech Co., Ltd., Foshan, 528138 Guangdong People’s Republic of China

**Keywords:** *Cystoisospora suis*, Enterotoxigenic *Escherichia coli*, Swine diseases, Co-infection, Diarrhea

## Abstract

**Background:**

While *Cystoisospora suis* is well established as a primary pathogen in suckling piglets, it can also infect weaned piglets. In this context, we investigated its co-infection with enterotoxigenic *Escherichia coli* (ETEC), a major cause of post-weaning diarrhea.

**Methods:**

Weaned piglets were randomly divided into four groups: a negative control group (NC), an ETEC single-infection group (EC), a *C. suis* single-infection group (CS), and a co-infection group (EC-CS). Following infection, clinical symptoms were recorded, and samples were collected to evaluate intestinal histopathological damage, expression of tight junction protein genes, inflammatory cytokine levels, and gut microbiota changes.

**Results:**

Compared to single-infection groups, piglets in the co-infection group exhibited more severe diarrhea, growth retardation, and intestinal damage, characterized by near-total loss of villus and crypt structures. Co-infection significantly impaired intestinal barrier function, as evidenced by a marked downregulation of *claudin-1* messenger RNA (mRNA) expression compared to both single-infection groups, and triggered more intense local and systemic inflammatory responses. 16S ribosomal RNA (rRNA) sequencing revealed that co-infection exacerbated gut microbiota dysbiosis and promoted the proliferation of pathogenic bacteria.

**Conclusions:**

Co-infection with *C. suis* and ETEC exerts a synergistic pathogenic effect in weaned piglets. The mechanism involves a vicious cycle of intestinal barrier disruption, microbiota dysbiosis, and amplified inflammatory responses. These findings provide a novel theoretical basis for the clinical prevention and control of complex intestinal co-infections.

**Graphical Abstract:**

**Figure:**
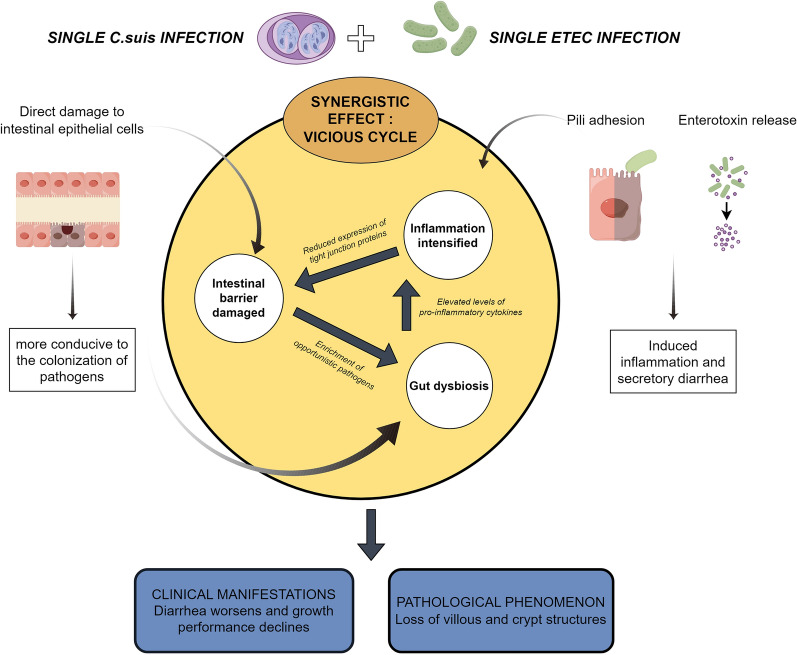

**Supplementary Information:**

The online version contains supplementary material available at 10.1186/s13071-026-07291-7.

## Background

Health management of piglets is a central link in the sustainable development of the global livestock industry, with direct implications for animal welfare, food safety, agricultural economic efficiency, and public health security. Diarrhea during the weaning period represents a critical bottleneck in intensive swine production. This condition is characterized by a complex etiology and substantial challenges in prevention and control, resulting in significant economic losses annually [1–4]. Research in this field is, therefore, of considerable importance.

Among the pathogens responsible for piglet diarrhea, *Cystoisospora suis* (*C. suis*) and enterotoxigenic *Escherichia coli* (ETEC) are two major pathogens capable of causing severe clinical outcomes [4–8]. *Cystoisospora suis* is an obligate intracellular protozoan parasite that disrupts intestinal epithelial integrity [9, 10], whereas ETEC adheres via fimbriae and secretes enterotoxins, resulting in secretory diarrhea [11–13]. Both pathogens colonize the small intestine, and their spatiotemporal coexistence creates a potential for strong interactions [6, 14]. Clinically, mixed infections involving these two gastrointestinal pathogens are common and are associated with more severe diarrhea, growth retardation, and increased mortality [15–17]. The high mortality associated with *C. suis* infection has been suggested to be linked to such mixed infections or secondary bacterial complications [14, 18–20]. However, current research focuses primarily on the pathogenic mechanisms or control measures of individual pathogens, leaving the synergistic pathogenesis during co-infection poorly understood.

A critical unresolved question is whether co-infection with these two pathogens exacerbates intestinal pathological damage or microbiota dysbiosis through synergistic effects. Does the disruption of the intestinal mucosal barrier by *C. suis* facilitate ETEC colonization? Conversely, does the inflammatory response induced by ETEC create a more favorable environment for *C. suis* proliferation? Addressing these questions is essential for developing precise prevention and control strategies. This study aims to experimentally validate the enhanced pathogenicity resulting from co-infection of *C. suis* and ETEC, thereby providing a scientific basis for the diagnosis and comprehensive management of clinical mixed infections.

## Methods

### Parasites and bacteria

#### Isolation and identification of *C. suis*

The *C. suis* isolate used in this study was originally obtained from a commercial pig farm in Guangdong Province, China, which had a documented history of neonatal diarrhea in suckling piglets. Clinical signs reported in the affected suckling piglets were consistent with *C. suis*, including yellowish, pasty diarrhea, and the condition showed poor response to antibiotic treatment. Microscopic examination confirmed the presence of *C. suis* oocysts in the feces. Fresh fecal samples were collected directly from the rectum of diarrheic suckling piglets. Oocysts were concentrated and isolated using the saturated sucrose solution flotation method. Genomic DNA was extracted from sporulated oocysts. The *ITS1* gene of *C. suis* was amplified by polymerase chain reaction (PCR), and the resulting amplicon was sequenced. BLAST analysis of the sequence confirmed that the isolated strain was *C. suis*. The strain is maintained and periodically passaged by Foshan Standard Bio-tech Co., Ltd.

#### Characterization of the ETEC strain

The ETEC strain (K88-2-JW) was first isolated in 2023 from weaned piglets suffering from severe post-weaning diarrhea on a pig farm in Guangdong Province, China. It was provided and preserved by Wen’s Foodstuffs Group Co., Ltd. The identification and virulence factor analysis of the strain were conducted according to standard PCR protocols described in previous studies [21, 22]. The results of virulence factors and serotyping for this strain are as follows: the K88 fimbrial encoding gene (*faeG*) was positive (K88+), while K99, F41, F18, and 987P fimbrial encoding genes were negative (K99−, F41−, F18−, 987P−). Regarding enterotoxin genes, the heat-stable enterotoxin b (STb) encoding gene (*estB*) was positive (STb+), but the heat-labile enterotoxin (LT) and heat-stable enterotoxin a (STa) encoding genes were negative (LT−, STa−). Additionally, the strain tested negative for Shiga toxin (Stx1 and Stx2) encoding genes, intimin (EAE) encoding gene (*eae*), enteroaggregative heat-stable enterotoxin (EAST1) encoding gene, porcine attaching and effacing-associated factor (PAA) encoding gene, and adhesin involved in diffuse adherence (AIDA) encoding gene, with the results being Stx1−, Stx2−, EAE−, EAST1+, PAA−, and AIDA−, respectively. O serotype identification confirmed it as O149-positive (O149+), verified by specific PCR amplification of the O149-related gene (*wzx*) [2]. Based on these findings, the isolate was defined as an ETEC of serotype O149:K88:STb. This serotype is frequently associated with post-weaning diarrhea in pigs according to the literature [22].

### Preparation of oocyst suspension and bacterial inoculum for piglet infection

#### Oocyst collection

Fecal samples containing *C. suis* oocysts were homogenized with distilled water and centrifuged at 3000 rpm for 5 min. The supernatant was discarded, and the pellet was resuspended in saturated sodium chloride solution. After mixing, the sample was centrifuged again at 3000 rpm for 5 min. The supernatant was collected and diluted with more than five volumes of distilled water. This mixture was centrifuged at 3000 rpm for 5 min, and the supernatant was discarded. The oocysts were transferred to a clean beaker, and an appropriate amount of 2.5% potassium dichromate solution was added. The beaker was placed on a shaker at 120 rpm and 26 °C for 24 h to obtain sporulated oocysts.

#### Oocyst purification

After collection, oocysts were purified using density gradient centrifugation. Briefly, TE [Tris–ethylenediaminetetraacetic acid (EDTA)] buffer was prepared by dissolving 1.51 g Tris and 0.92 g EDTA in 250 ml of sterile water. A stock solution was made by dissolving 3.79 g cesium chloride in 18 ml TE buffer. Gradient solution A was prepared by mixing 4 ml stock solution with 6 ml TE buffer; gradient solution B by mixing 6 ml stock solution with 4 ml TE buffer and adding 0.001 g food red; and gradient solution C by mixing 8 ml stock solution with 2 ml TE buffer. The sporulated oocysts were placed in a centrifuge tube, centrifuged at 3000 rpm for 5 min, and the supernatant discarded. The pellet was resuspended in an appropriate amount of TE buffer. Using a 5-ml syringe, 3 ml of gradient solution A was slowly added to the bottom of a 15-ml centrifuge tube. Then, with a long needle, 3 ml of gradient solution B was injected slowly into the bottom, followed by 3 ml of gradient solution C using the same method. The tube was centrifuged at 4 °C and 12,000×*g* for 60 min. Pure oocysts were collected from the interface between gradient solutions A and B. The collected fraction was transferred to another tube, diluted with more than five volumes of distilled water, and centrifuged at 4 °C and 3000 rpm for 5 min. The supernatant was discarded to obtain purified oocysts.

#### Removal of fecal microbial contamination from oocyst suspension

Density gradient centrifugation effectively separated oocysts from other impurities, including bacteria and viruses. After purification, a 2.5% potassium dichromate solution filtered through a 0.22-μm membrane was added to the purified oocysts to prevent pathogen growth. The mixture was sealed and stored at 4 °C. Before infecting piglets, the oocyst suspension was subjected to sterility testing on various culture media and quantitative fluorescence PCR for exogenous pathogens.

#### Oocyst counting

Purified oocysts were counted using a hemocytometer. The oocyst suspension was thoroughly mixed with a pipette, and 10 μl was added to each chamber of the counting slide. The total number of oocysts in the four large squares at each corner of the chamber was recorded as one count; this was repeated for 10 counts. The highest and lowest values were excluded, and the mean of the remaining eight counts was calculated. This mean was divided by 4 (representing the four squares) to obtain *x*, where *x* represents the concentration in 10,000/ml. Before infection, the oocyst suspension was centrifuged and resuspended in sterile water to adjust the concentration to 5000 oocysts/ml.

#### Amplification of ETEC and preparation of challenge inoculum

Bacterial stocks stored at −80 °C in glycerol were streaked onto Luria–Bertani (LB) agar medium (Huankai Microbial, China) and incubated at 37 °C for 16–18 h. A single colony was picked and inoculated into 5 ml of tryptic soy broth (Becton, Dickinson and Company [BD], USA), followed by shaking at 200 rpm and 37 °C for 12 h to prepare a seed culture [13]. The seed culture was transferred at a 1:100 ratio into fresh tryptic soy broth (BD, USA) and incubated under the same conditions until the logarithmic growth phase (OD_600_ ≈ 0.6). The bacteria were harvested by centrifugation at 4 °C and 5000×*g* for 15 min. The pellet was washed twice with sterile phosphate-buffered saline (PBS) and resuspended in sterile PBS.

#### Standardization of challenge inoculum and infection dose

Viable bacterial counts were determined by the plate count method. The bacterial suspension was serially diluted 10-fold, and 100 μl of an appropriate dilution was spread onto LB agar medium (Huankai Microbial, China). After overnight incubation at 37 °C, colony-forming units (CFU) were counted. The suspension was adjusted with sterile PBS to a target concentration of 1 × 10^9^ CFU/ml. The challenge inoculum was kept on ice and administered orally to piglets within 2 h of preparation, with each piglet receiving 1 ml (i.e., 1 × 10^9^ CFU per animal).

### Animal infection experiments

**Fig. 1:**
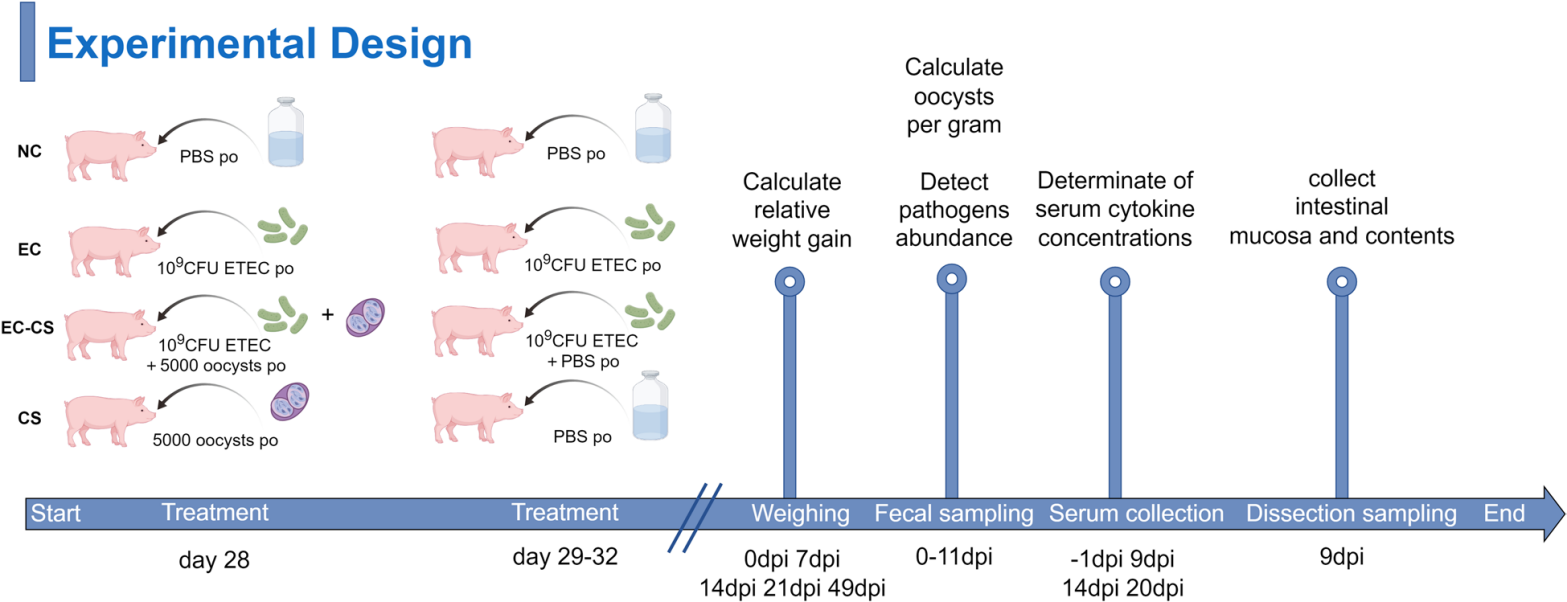
Schematic of the experimental animal grouping and infection timeline (by FigDraw)

Three-week-old female Duroc × (Landrace × Yorkshire) three-way cross hybrid piglets were obtained from a healthy, *C. suis*-negative herd in Yunfu, Guangdong Province. Only female piglets were used in order to minimize variability influenced by sex differences and to reduce aggression in group housing. A total of 28 weaned piglets from eight different healthy litters were selected for the trial at 21 days of age. Continuous monitoring with twice-weekly sampling after birth ensured that all experimental animals tested negative for ETEC, *C. suis*, *Clostridium perfringens*, Shiga toxin-producing *E. coli* (STEC), *Lawsonia intracellularis*, porcine epidemic diarrhea virus (PEDV), and porcine rotavirus (PoRV) prior to infection. At 21 days of age, the 28 piglets were randomly assigned to four groups (*n* = 7 per group) using a computer-generated random number sequence. To minimize the potential impact of litter effects, piglets from the same litter were distributed as evenly as possible across the different groups. No single group contained more than two piglets from the same litter. The groups were as follows: negative control (NC), ETEC single-infection (EC), ETEC and *C. suis* co-infection (EC-CS), and *C. suis* single-infection (CS). One week later (at 28 days of age), piglets in the CS and EC-CS groups were orally inoculated with 1 ml of *C. suis* Guangdong strain (5000 sporulated oocysts/ml). From days 28 to 32 of age, piglets in the EC and EC-CS groups received daily oral inoculations of 1 ml of ETEC (1 × 10^9^ CFU/ml) (as outlined in Fig. 1). The NC group received an equivalent volume of PBS orally at the same time points. Infection methods, doses, and intervals for both pathogens were based on previous studies [19, 23–28] and pre-experimental trials, with minor adjustments for practical conditions. All time points are reported relative to the *C. suis* infection; for instance, 1 day post-infection (dpi) corresponds to day 29 of age. The body weight of all piglets was measured at 0 dpi, and no significant difference in the initial body weight was observed among the four groups (*P* > 0.05, one-way analysis of variance [ANOVA]), ensuring a comparable baseline for subsequent analysis. To assess the pathological effects during the acute phase of infection while retaining a sufficient number of animals for the subsequent production performance, three piglets per group were randomly selected for necropsy and sampling at 9 dpi. The remaining piglets were maintained for continuous monitoring of body weight, diarrhea scores, and other parameters throughout the study period.

### Clinical evaluation

Clinical assessment included body weight, fecal score (FS), and mortality rates [18, 26, 29–31]. Following *C. suis* infection, FS was evaluated daily from 5 to 11 dpi. The scoring system was as follows: FS 1, firm; FS 2, pasty; FS 3, semi-liquid; FS 4, liquid (with FS 3 and FS 4 defined as diarrhea) [27, 29]. Body weight was monitored weekly throughout the study period (0–49 dpi). Survival rates and general health status were recorded daily.

### Oocysts per gram of feces

Fecal samples were collected prior to *C. suis* infection and from 0 to 11 dpi for oocyst quantification (oocysts per gram, OPG) [32]. After thorough homogenization, 3 g of feces was mixed with 42 g of flotation solution and filtered through gauze [33]. The suspension was transferred into two chambers of a McMaster counting slide (Shanghai Veterinary Research Institute, China) and allowed to settle for 5 min. Oocysts were counted, and the OPG value was calculated based on counts from both chambers [27, 34, 35].

### DNA extraction and pathogen load detection

Fecal samples (0.5 g) were homogenized in 2 ml PBS. Intestinal content samples were weighed, and 1 ml PBS was added before grinding. Intestinal mucosa samples were weighed, incubated with 1 ml of 1% trypsin (Gibco, USA) for 1 h, and then ground. A 400-μl aliquot was processed using a nucleic acid extraction kit (Biaoyun Biology, China) on an automated nucleic acid extraction system (Bioer, China). Extracted nucleic acids served as the template for quantitative PCR (qPCR) to determine *C. suis* fecal shedding titers [36, 37]. A standard curve was generated from serial dilutions of a plasmid of known concentration, and pathogen load (copies/ml) was calculated accordingly. The average pathogen load per group was determined from the mean cycle threshold (Ct) of positive samples. Primer sequences are listed in Table 1 [38]. The primers and probe for ETEC detection were designed to target the STb encoding gene, based on sequence AY028790.

**Table 1:** Primer sequences used for quantitative real-time PCR (qRT-PCR)

Gene name	Primer sequence (5′ → 3′)​	Source or reference
*C. suis*	Forward: CGGTAGCGGTACCCTTCATG	[38]
	Reverse: CCCGCACAGAACACAAACG	[38]
	Probe: CTTCTCTGCATCTCTC	[38]
ETEC	Forward: GCCCAAATAATGGTTGCAG	This study
	Reverse: GCGTTAGGACATTGTCAC	This study
	Probe: ATAGCATTCAGCACCATATACACA	This study
*Zo-1*	Forward: AGCCCGAGGCGTGTTT	[64]
	Reverse: GGTGGGAGGATGCTGTTG	[64]
*occludin*	Forward: GCACCCAGCAACGACAT	[64]
	Reverse: CATAGACAGAATCCGAATCAC	[64]
*claudin-1*	Forward: GACTCCTTGCTGAATCTGA	[64]
	Reverse: GCACCTCATCATCTTCCAT	[64]
*GAPDH*	Forward: ACTCACTCTTCCACTTTTGATGCT	This study
	Reverse: TGTTGCTGTAGCCAAATTCA	This study
*IL-1β*	Forward: CTGTGTGGGGCCTGACAA	[64]
	Reverse: AGTGCTTGCAGTCGAACTCA	[64]
*IL-10*	Forward: CCAGAGGTCCGACCACTACA	[64]
	Reverse: GGTCCCCTTCAATCCTGTTGAA	[64]
*CXCL10*	Forward: ATCAAGCCCTAACTGTCCATG	This study
	Reverse: AATGTAGCACCTCAGTGTAGC	This study

### Cytokine detection

Whole blood was collected from piglets in each group at −1, 9, 14, and 20 dpi, and serum was prepared. Serum levels of interleukin (IL)-6, IL-12, IL-2, IL-1β, interferon-γ (IFN-γ), tumor necrosis factor-α (TNF-α), and IL-10 were quantified using commercial enzyme-linked immunosorbent assay (ELISA) kits (mlbio, China) according to the manufacturer’s instructions [26, 29, 31].

### Histopathological analysis

Jejunal and ileal tissue specimens were fixed in tissue fixative (Servicebio, China) at room temperature for 24 h, paraffin-embedded, sectioned, and stained with hematoxylin and eosin (H&E). Intestinal morphology was examined by light microscopy. For each section, five intact and representative villi were selected to measure villus height (VH) and crypt depth (CD). The VH/CD ratio was calculated and analyzed [23, 39].

### 16S rRNA sequencing

Intestinal contents (jejunum, ileum, and cecum) were collected, placed in cryovials, and immediately flash-frozen in liquid nitrogen. The cecum was included, as it is a major site of microbial fermentation with high diversity [40], and its microbiota is a well-established sensor for overall gut health status in pigs [41–43]. Samples were shipped on dry ice to Suzhou Azenta Company for 16S ribosomal RNA (rRNA) gene sequencing and microbiota analysis [29, 31, 44, 45].

### Measurement of gene expression

At 9 dpi, jejunal samples from the distal region of three piglets per group were collected for RNA extraction. Total RNA was isolated using an RNA extraction kit (Beyotime, China) and reverse-transcribed to complementary DNA (cDNA) using a reverse transcription kit (Beyotime, China). Quantitative real-time PCR (qRT-PCR) was performed to determine expression levels, normalized to the reference gene *GAPDH*, and expressed as fold changes relative to the NC group. Primer sequences are listed in Table 1 [38, 64].

### Statistical analysis

All data were analyzed and visualized using R software, and presented as mean ± standard error of the mean (SEM). Differences between means were assessed using *t*-tests, two-way ANOVA, and Tukey’s test, with *P* < 0.05 considered statistically significant. Asterisks denote significance levels: *P < 0.05, **P < 0.01, ***P < 0.001, and ****P < 0.0001.

## Results

### Co-infection with *C. suis* and ETEC exacerbates clinical symptoms and intestinal pathological damage in weaned piglets

**Fig. 2:**
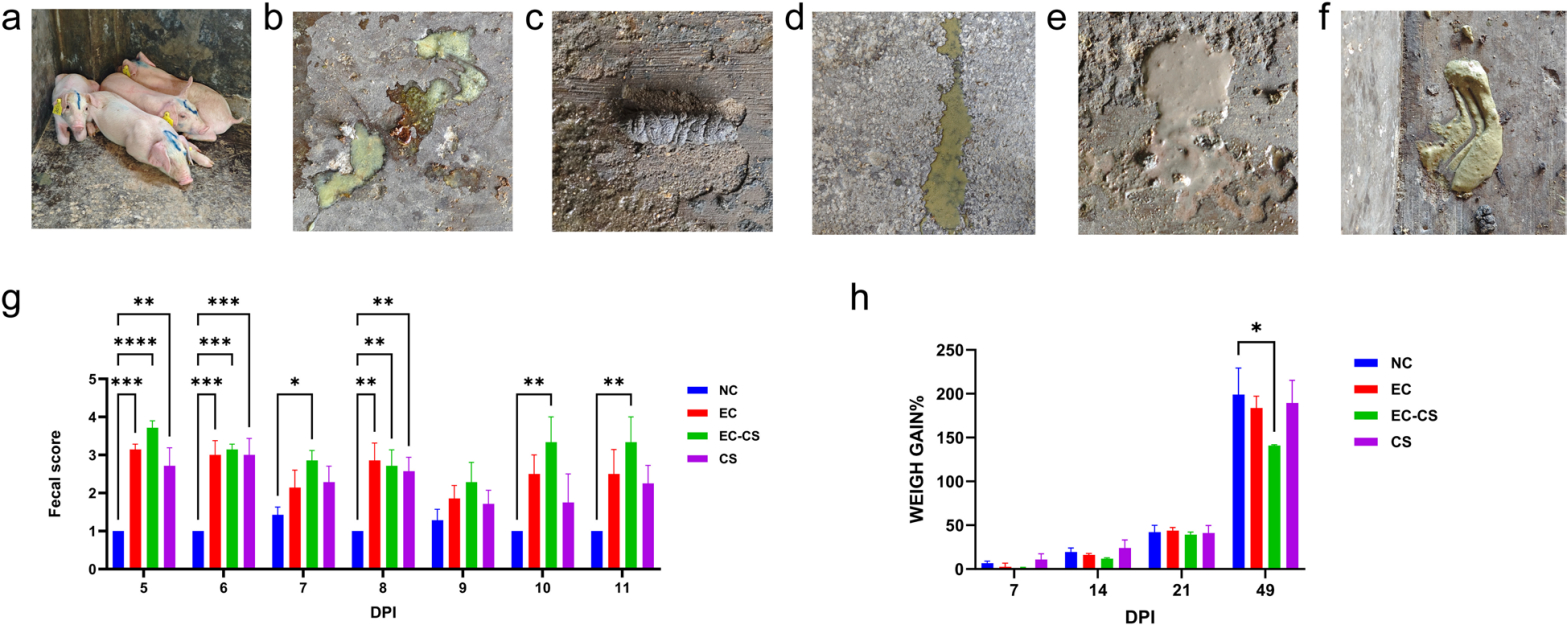
Co-infection with *C. suis* and ETEC exacerbates clinical signs in weaned piglets. **a** Mental state (depression) observed in the EC-CS group following infection. **b** Vomitus observed in the EC and EC-CS groups. **c–f** Representative fecal samples from the NC, EC, EC-CS, and CS groups, respectively. **g** Fecal scores at 5–11 dpi. Data are presented as mean ± SEM (*n* = 7 per group initially; after 9 dpi, *n* = 4 for NC, EC, and CS groups; *n* = 3 for the EC-CS group, following mortality). **h** Mean body weight of piglets throughout the experimental period. Data are presented as mean ± SEM (*n* = 4 per group initially; after 9 dpi, *n* = 4 for NC, EC, and CS groups; *n* = 3 for the EC-CS group, following mortality). Asterisks indicate significant differences between groups: *P < 0.05; **P < 0.01; ***P < 0.001

Piglets in the NC group remained mentally alert and exhibited no significant changes in appetite. In contrast, piglets in the three infected groups displayed lethargy, anorexia, and an increased tendency to lie down (Fig. 2a). Mortality occurred exclusively in the co-infected (EC-CS) group at 8 dpi. Vomiting was observed in the EC and EC-CS groups during the early stage of infection (Fig. 2b). Of note, vomiting has been documented as a possible symptom of ETEC infection [46]. Furthermore, to rule out involvement of other common enteric pathogens, fecal samples from all piglets were tested at 6 dpi for PEDV, PoRV, *C. perfringens*, STEC, and *L. intracellularis*, all with negative results. Fecal consistency was normal in the NC group (Fig. 2c), whereas all infected groups developed diarrhea of varying severity. Specifically, the EC group produced yellow, liquid feces (Fig. 2d), the EC-CS group had brown, liquid feces (Fig. 2e), and the CS group passed yellow-white, pasty feces (Fig. 2f). From 5 to 11 dpi, diarrhea was absent in the NC group, but FS values were significantly elevated in all three infected groups compared to the NC controls (Fig. 2g). Notably, the EC-CS group consistently showed the highest FS values across most time points, with the exception of 8 dpi.

Body weight monitoring over 7 weeks revealed that the relative weight gain rate in the EC-CS group was significantly lower than that in the NC group (ANOVA, *F*_(3, 44)_ = 1.709, *P* = 0.0134), indicating that co-infection led to more severe impairment of growth performance (Fig. 2h).

**Fig. 3:**
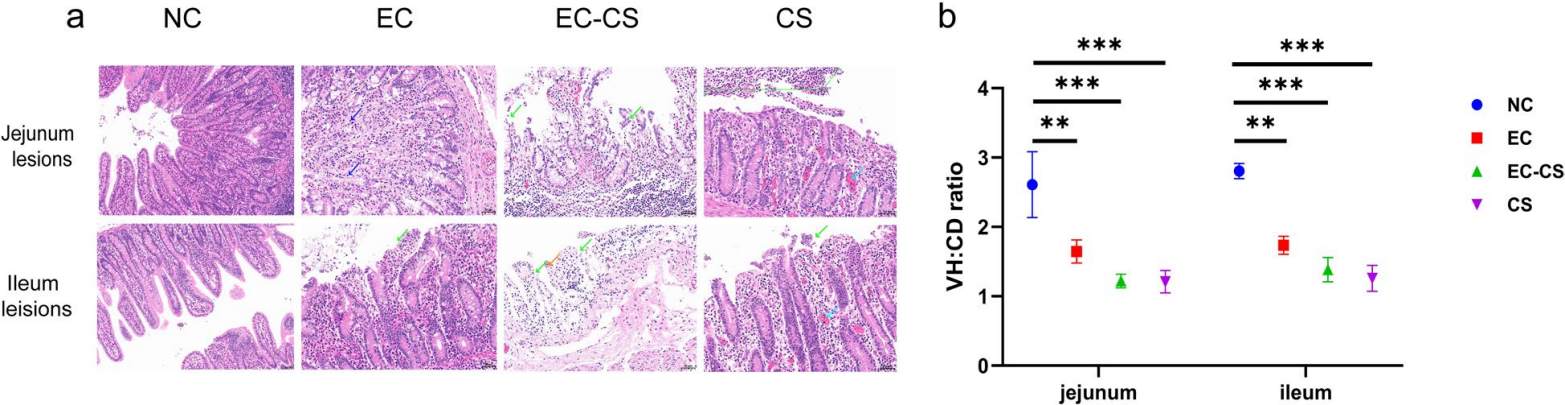
Co-infection with *C. suis* and ETEC exacerbates intestinal pathological damage in piglets. **a** Histopathological sections in intestinal tissues from different groups. The EC and CS groups exhibited epithelial cell detachment (green arrows) and inflammatory cell infiltration (blue arrows). Scale bar: 50 µm. **b** Villus height to crypt depth ratio in the intestine of piglets from different groups. Asterisks indicate significant differences between groups: *P < 0.05; **P < 0.01; ***P < 0.001

Histopathological analysis of jejunal and ileal sections (Fig. 3a) showed normal villus morphology, high density, and an intact epithelium in the NC group. The EC group exhibited extensive epithelial cell detachment, localized erosion, and crypt loss in the posterior jejunum, while the ileum showed slight epithelial shedding, exposed lamina propria, and a reduced number of goblet cells. The most severe damage was observed in the EC-CS group, where both the ileum and posterior jejunum displayed widespread villous epithelial cell detachment, exposed lamina propria, near-total loss of villous and crypt structures, and a marked decrease in goblet cells. In the CS group, the posterior jejunum and ileum showed extensive epithelial cell detachment, exposed lamina propria, reduced goblet cells, and scant luminal leukocytes and necrotic debris. Consistent with these observations, the VH/CD ratio was significantly reduced in the posterior jejunum and ileum of the EC-CS group compared to the NC group (ANOVA, *F*_(3, 16)_ = 57.86, *P* < 0.0001; Fig. 3b).

### Co-infection with *C. suis* and ETEC exacerbates intestinal barrier dysfunction​ in weaned piglets

**Fig. 4:**
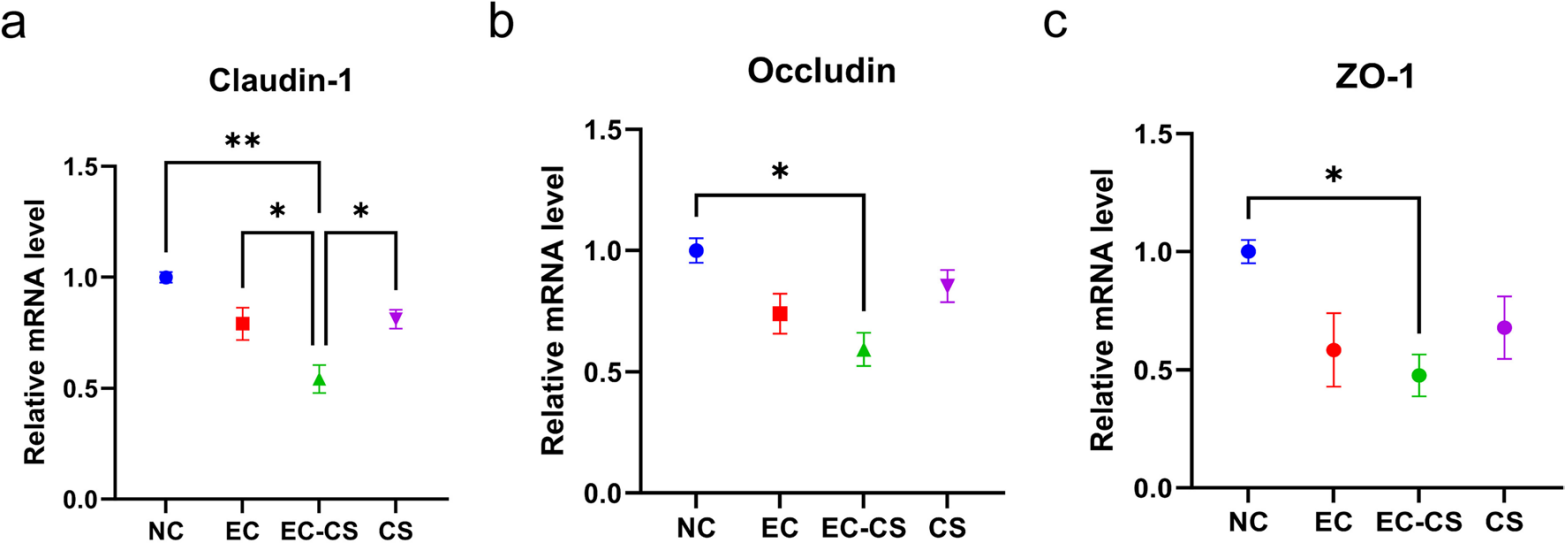
Co-infection with *C. suis* and ETEC exacerbates the impairment of intestinal barrier function. **a–c** Relative mRNA expression levels of tight junction proteins *claudin-1*, *occludin*, and *ZO-1* genes in the jejunal mucosa, as determined by qRT-PCR. Data are presented as mean ± SEM (*n* = 3). Asterisks indicate significant differences between groups: *P < 0.05; **P < 0.01; ***P < 0.001

Co-infection significantly impaired intestinal barrier function, as evidenced by the downregulation of key tight junction proteins. Compared to the NC group, the EC-CS group showed significantly lower relative messenger RNA (mRNA) expression of *claudin-1* (ANOVA, *F*_(3, 8)_ = 12.13, *P* = 0.0015; Fig. 4a), *occludin* (ANOVA, *F*_(3, 8)_ = 6.435, *P* = 0.0122; Fig. 4b), and *ZO-1* (ANOVA, *F*_(3, 8)_ = 3.906, *P* = 0.0474; Fig. 4c) in jejunal tissue. Notably, *claudin-1* expression was also significantly lower in the EC-CS group than in either the EC group (ANOVA, *F*_(3, 8)_ = 12.13, *P* = 0.0459) or the CS group (ANOVA, *F*_(3, 8)_ = 12.13, *P* = 0.0315) (Fig. 4a). These results indicate that while all three infection modes disrupted the intestinal barrier, co-infection resulted in the most severe impairment.

### Co-infection with *C. suis* and ETEC triggers and amplifies systemic and intestinal inflammatory responses

**Fig. 5:**
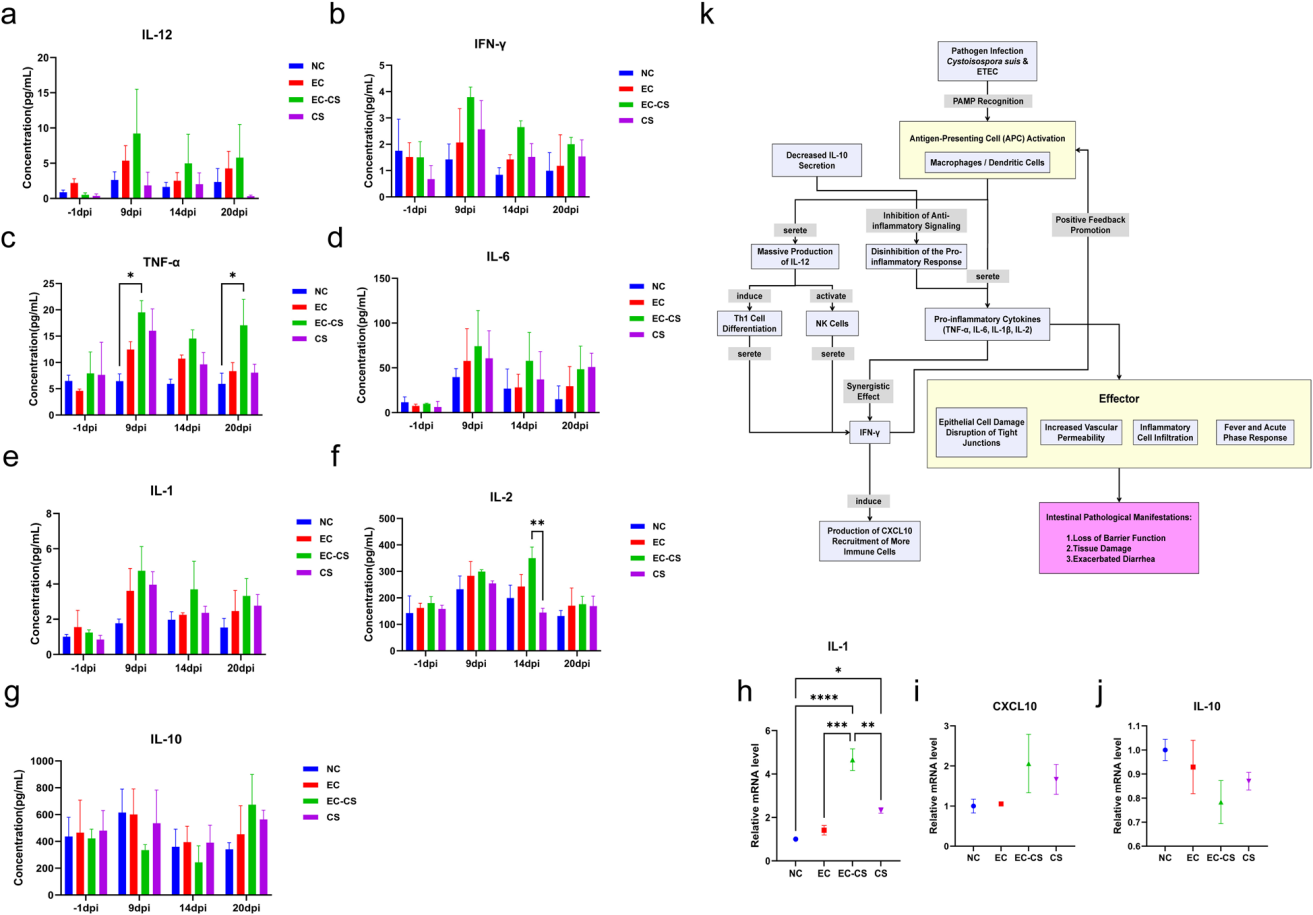
Systemic and intestinal pro-inflammatory responses are heightened in co-infected piglets. **a–f** Serum concentration changes in key pro-inflammatory cytokines (IL-12, IFN-γ, TNF-α, IL-6, IL-1β, and IL-2). **g** Serum concentration of the anti-inflammatory cytokine IL-10. **h–j** Relative mRNA expression levels of *IL-1*, *CXCL10*, and *IL-10* in the jejunal mucosa, as determined by qRT-PCR. **k** Proposed interaction network of immune mediators during co-infection with *C. suis* and ETEC. Data are presented as mean ± SEM (*n* = 3). Asterisks indicate significant differences between groups: *P < 0.05; **P < 0.01; ***P < 0.001; ****P < 0.0001

Serum levels of pro-inflammatory cytokines (IL-12, IFN-γ, TNF-α, IL-6, IL-1β, and IL-2) and the anti-inflammatory cytokine IL-10 were measured using ELISA. As shown in Fig. 5a–f, infection generally increased the levels of IL-12, IFN-γ, TNF-α, IL-6, IL-1β, and IL-2 across all infected groups. Specifically, TNF-α levels in the EC-CS group were significantly higher than those in the NC group (ANOVA, F_(3, 32_) = 6.491, *P* = 0.0121 and *P* = 0.0400, respectively) at 9 and 20 dpi. Additionally, IL-2 concentrations in the EC-CS group were significantly elevated compared to the NC group (ANOVA, *F*_(3, 32)_ = 3.156, *P* = 0.0474) and the CS group (ANOVA, *F*_(3, 32)_ = 3.156, *P* = 0.0039) at 14 dpi. In most instances, the EC-CS group exhibited the highest levels of IL-12, IFN-γ, TNF-α, IL-6, IL-1β, and IL-2. Concurrently, the anti-inflammatory cytokine IL-10 was downregulated post-infection (at 9 and 14 dpi) (Fig. 5g), indicating the most severe systemic inflammatory response in the EC-CS group.

In the jejunal mucosa, the relative mRNA expression of *IL-1* was significantly upregulated in the EC-CS group and CS group compared to the NC group (ANOVA, *F*_(3, 8)_ = 34.32, *P* < 0.0001 and *P* = 0.0414; Fig. 5h). Furthermore, *IL-1* expression in the EC-CS group was significantly higher than in either the EC group (*P* = 0.0002) or the CS group (*P* = 0.0016), indicating the most intense intestinal inflammatory response under co-infection. The expression of the C-X-C motif chemokine ligand 10 (*CXCL10*) was also markedly increased in the EC-CS and CS groups (Fig. 5i). In contrast, *IL-10* mRNA expression was reduced in all infected groups, with the most pronounced decrease observed in the EC-CS group (Fig. 5j).

To elucidate the immunological mechanisms underlying severe intestinal damage induced by co-infection, we constructed a pathway map based on measured immune factor levels (Fig. 5k). Co-infection triggered a series of synergistically amplified immune responses: pathogen-associated molecular patterns were recognized by antigen-presenting cells (e.g., macrophages and dendritic cells), leading to their activation. Activated antigen-presenting cells produced substantial IL-12, which promoted T helper 1 (Th1) cell differentiation and natural killer (NK) cell activation. These cells, in turn, secreted high levels of IFN-γ, which further activated antigen-presenting cells via a positive feedback loop and induced CXCL10 production, recruiting additional immune cells to the intestine and exacerbating inflammation. Meanwhile, pro-inflammatory cytokines (TNF-α, IL-6, IL-1β, IL-2) were abundantly released, whereas the anti-inflammatory cytokine IL-10 was suppressed, resulting in an imbalance between pro- and anti-inflammatory signals. This dysregulated immune state acted on downstream effectors, causing intestinal epithelial cell damage, disruption of tight junctions, and increased vascular permeability, ultimately leading to tissue injury, compromised barrier function, and aggravated diarrhea. These results demonstrate at the molecular level that co-infection synergistically drives immune dysregulation, characterized by a dominant Th1-type response and uncontrolled inflammatory signaling, which constitutes a key mechanism of severe intestinal pathology in weaned piglets.

### Co-infection with *C. suis* and ETEC induces intestinal microbiota dysbiosis and promotes pathogen proliferation

**Fig. 6:**
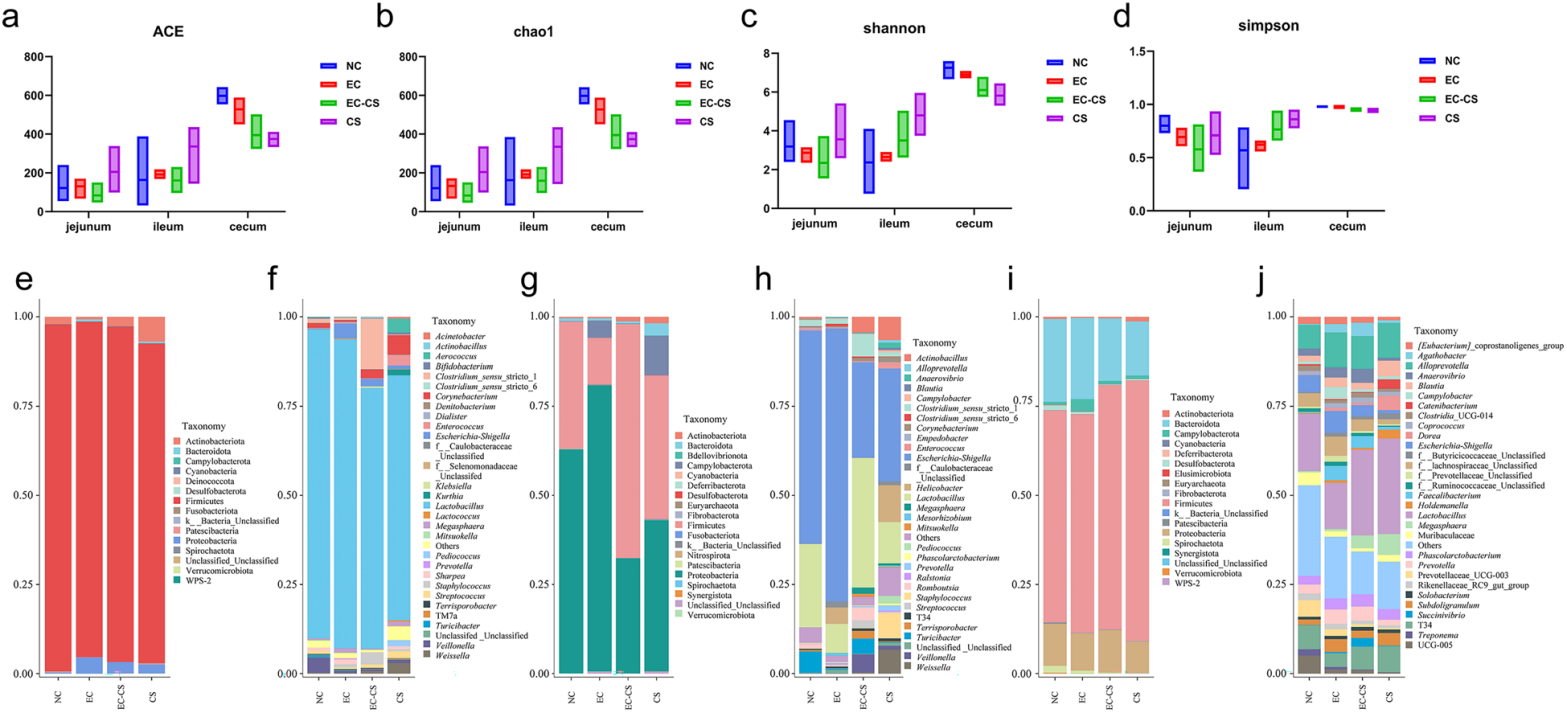
Co-infection with *C. suis* and ETEC alters the gut microbial community structure in weaned piglets. **a**, **b** Alpha diversity indices reflecting microbial richness (ACE and Chao1) across different intestinal segments. **c**, **d** Alpha diversity indices reflecting microbial diversity (Shannon and Simpson). **e**, **f** Microbial composition in jejunal contents at the phylum (**e**) and genus (**f**) levels. **g**, **h** Microbial composition in ileal contents at the phylum (**g**) and genus (**h**) levels. **i**, **j** Microbial composition in cecal contents at the phylum (**i**) and genus (**j**) levels

Alpha diversity analysis of the microbial community, based on sequencing results of jejunal, ileal, and cecal contents, is shown in Fig. 6a, b. The species richness indices (ACE [Abundance-based Coverage Estimator] and Chao1) were significantly reduced in the EC-CS group compared to the NC group across all intestinal segments. In most cases, these values were the lowest among all groups, indicating that co-infection substantially compromised the species richness of the intestinal microbiota in piglets. Furthermore, the diversity indices (Shannon and Simpson) demonstrated that the NC group exhibited the highest values in the jejunum and cecum, suggesting greater evenness, lower dominance, and higher diversity of the microbiota in these regions (Fig. 6c, d). In contrast, the EC-CS group showed a marked decline in diversity levels, indicating severe impairment of microbial diversity and evenness. The EC group showed an intermediate state between the NC and EC-CS groups.

Analysis of the microbial community composition in jejunal contents at the phylum level (Fig. 6e) revealed that Firmicutes, Actinobacteriota, and Proteobacteria were the dominant phyla in all groups. Compared to the NC group, the most notable change in the three infected groups was a significant increase in the relative abundance of Proteobacteria, which includes various opportunistic pathogens (e.g., *E. coli* belongs to this phylum). An elevated proportion of Proteobacteria is a classical hallmark of intestinal microbiota dysbiosis. Additionally, the relative abundance of Actinobacteriota was markedly elevated in the CS group. At the genus level (Fig. 6f), *Lactobacillus* was the predominant genus across all groups. The EC group exhibited an increase in the abundance of *Escherichia-Shigella* (4.16%, the highest among groups). In the EC-CS group, the abundance of the probiotic *Lactobacillus* decreased, whereas that of the potential pathogen *Clostridium* sensu stricto 1 increased to 13.95%, substantially exceeding other groups, suggesting that co-infection may significantly promote the proliferation of this genus. Moreover, the abundance of other opportunistic pathogens, such as *Escherichia-Shigella*, *Corynebacterium*, *Staphylococcus*, and *Streptococcus*, were elevated to varying degrees.

Analysis of the microbial community composition in ileal contents at the phylum level (Fig. 6g) identified Firmicutes and Proteobacteria as the dominant phyla. In the EC group, Proteobacteria became the predominant phylum, with an average relative abundance of 80.25%, while Firmicutes decreased markedly to 12.94%. A slight increase was also observed in the abundance of Campylobacterota. In the EC-CS group, Firmicutes was the most abundant phylum (65.37%), and the relative abundance of Proteobacteria declined to 32.32%. In the CS group, the proportions of Proteobacteria and Firmicutes were comparable, and the relative abundance of Campylobacterota increased to 11.00%, significantly higher than in other groups. At the genus level (Fig. 6h), *Escherichia-Shigella* became the dominant genus in the EC group, accounting for the increase in Proteobacteria. In contrast, *Lactobacillus* was severely suppressed, while other genera such as *Helicobacter* exhibited a moderate increase (4.59%). The EC-CS group displayed considerable restructuring of the microbial community, with high interindividual variability. Different samples were dominated by *Escherichia-Shigella*, *Clostridium* s.s. 1, or *Lactobacillus*, indicating that co-infection may destabilize the ileal environment, leading to stochastic microbial colonization outcomes. In the CS group, *Helicobacter* was markedly enriched, with a mean relative abundance of 10.32%, substantially higher than in other groups, explaining the surge of Campylobacterota at the phylum level.

Analysis of cecal content sequencing at the phylum level (Fig. 6i) showed that the cecal microbiota was primarily composed of Firmicutes and Bacteroidota. In the EC group, the Firmicutes/Bacteroidota (F/B) ratio was similar to that in the NC group; however, interindividual variability increased, with some samples showing elevated levels of Proteobacteria and Campylobacterota. In the EC-CS group, the relative abundance of Bacteroidota decreased while that of Proteobacteria increased to 12.02%. The CS group exhibited strong suppression of Bacteroidota, resulting in the lowest relative abundance among the groups. At the genus level (Fig. 6j), in the EC group, opportunistic pathogens such as *Escherichia-Shigella* (6.16%) and *Campylobacter* (3.39%) showed increased relative abundance compared to the NC group, whereas the probiotic *Lactobacillus* decreased to 12.96%, suggesting that the ETEC infection model may facilitate the proliferation of potential pathogens while inhibiting beneficial bacteria. The CS group differed significantly from the EC group, with elevated relative abundance of *Megasphaera* and *Streptococcus* and a reduction in *Escherichia-Shigella*, implying that *C. suis* infection influences microbial structure through distinct mechanisms. In the EC-CS group, microbial changes exhibited an intermediate trend between EC and CS, with the relative abundance of *Escherichia-Shigella* lower than in the EC group but higher than in the CS group.

**Fig. 7:**
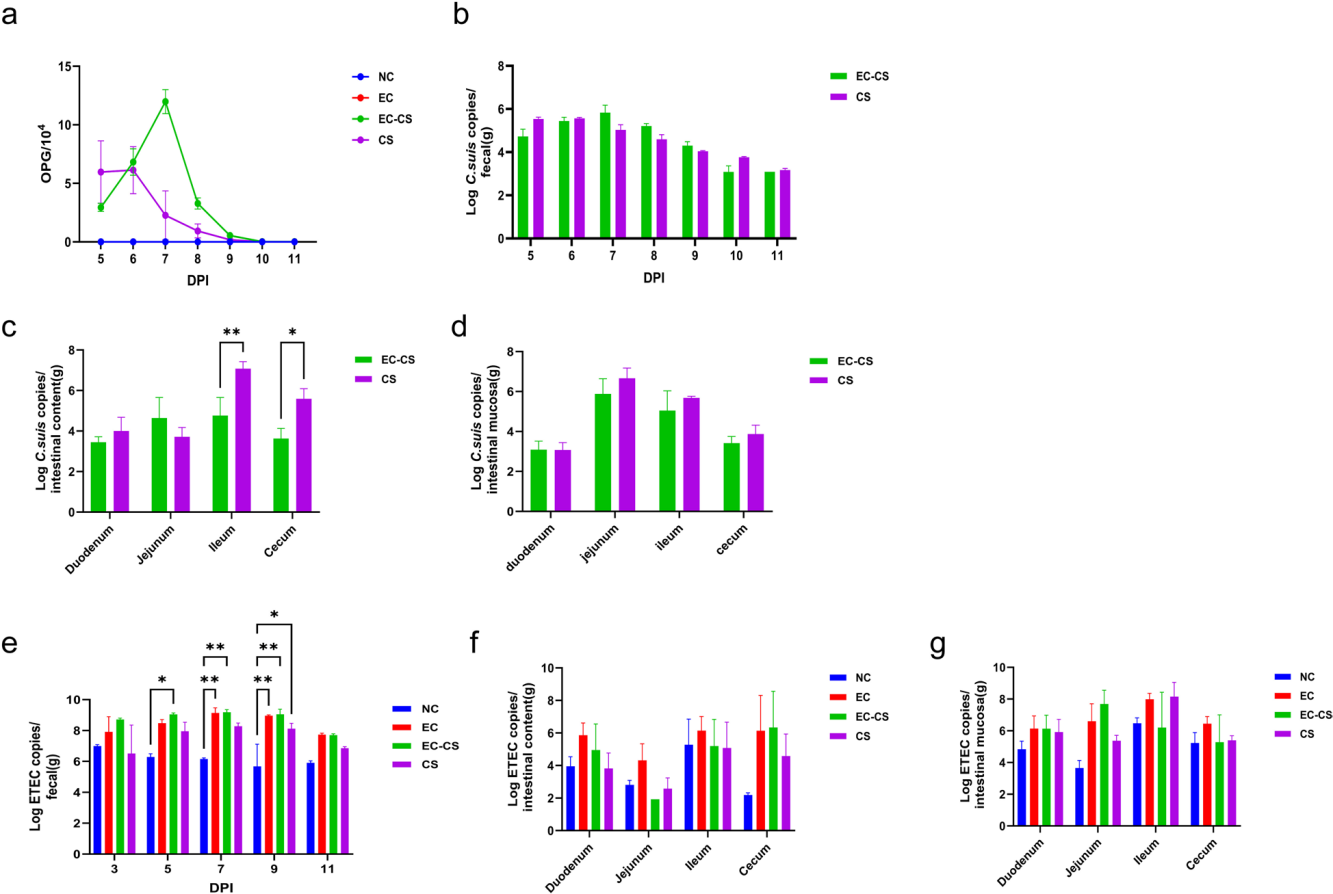
Pathogen load of *C. suis* and ETEC in feces, intestinal contents, and mucosa of piglets across experimental groups. **a** Oocysts per gram (OPG) of feces in the EC-CS group and CS group. **b** *Cystoisospora suis* load in feces from 5 to 11 days post-infection (dpi). **c**, **d** *Cystoisospora suis* load in intestinal contents and mucosa at 9 dpi. **e** ETEC load in feces at 5–11 dpi. **f**, **g** ETEC load in intestinal contents and mucosa at 9 dpi

Coccidial monitoring indicated that no oocysts were excreted in any groups before challenge. Oocyst excretion began at 5 dpi in the EC-CS and CS groups. The oocyst excretion patterns for each group are shown in Fig. 7a. The CS group displayed a peak in oocyst excretion at 5–6 dpi, while the EC-CS group peaked at 7 dpi, representing a delay of 1–2 days compared to the CS group. However, the oocyst quantity at the peak was higher in the EC-CS group than in the CS group. No oocysts were detected in the NC or EC groups during the experimental period. Quantitative PCR analysis of microscopy-positive fecal samples yielded results consistent with microscopic observations (Fig. 7b). Compared to the EC-CS group, the CS group had a significantly higher coccidial load at 9 dpi (in the contents of the ileum and cecum, and the mucosa of the jejunum, ileum, and cecum; Fig. 7c, d). This phenomenon may be attributed to the earlier onset of the oocyst excretion peak in the CS group relative to the EC-CS group. Following a second ingestion of oocysts, most coccidia in the CS group completed their life cycle earlier, being excreted from the jejunal and ileal mucosa into the contents and migrating to the ileum and cecum for eventual fecal excretion. ETEC monitoring demonstrated an increase in fecal bacterial excretion across all three infected groups, with loads ranging from 10^6.5^ to 10^9.05^ copies/g. At 3 and 5 dpi, the EC-CS group showed higher bacterial excretion levels than the EC group; however, these levels subsequently equilibrated (Fig. 7e). Throughout the trial, *C. suis* and ETEC tested negative in the NC group and in all piglets before infection. At 9 dpi, the EC group demonstrated a higher ETEC load in the intestine compared to the EC-CS group (Fig. 7f, g). This difference might be because ETEC is acting as the sole dominant bacterium in the EC group’s intestine, whereas in the EC-CS group, the complexity of the gut environment, potentially involving proliferation of other opportunistic pathogens, hindered ETEC from becoming the exclusive dominant bacterium.​

## Discussion

**Fig. 8:**
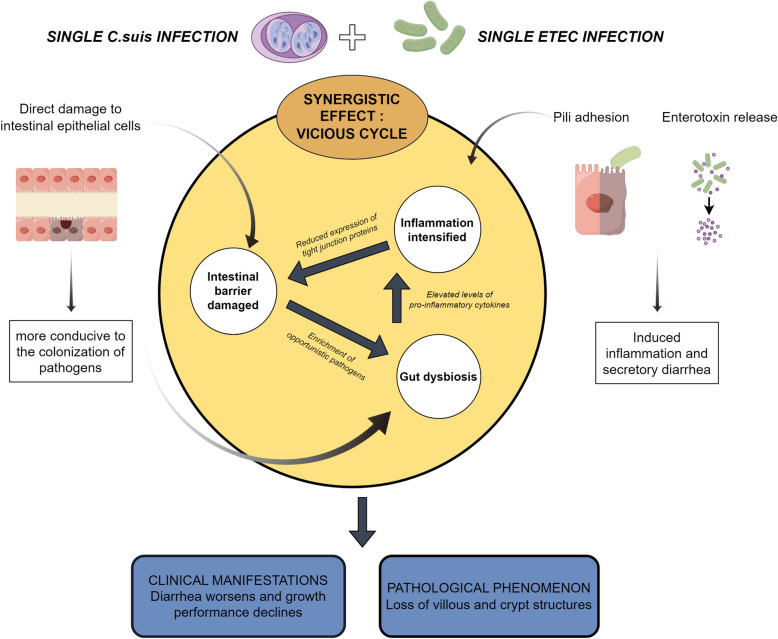
Proposed model illustrating the synergistic mechanisms of intestinal pathogenicity potentiated by co-infection with *C. suis* and ETEC (by FigDraw)

Digestive tract co-infections involving parasites and bacteria are a significant cause of high morbidity and mortality in animals [20, 47]. The pathogenesis of such co-infections is characterized by complex interactions between pathogens and the host, physical barrier disruption, and immune response dysregulation [48], which can collectively enhance pathogen proliferation, exacerbate tissue damage, and increase disease incidence. Clinically, concurrent infection with *C. suis* and ETEC is frequently observed. In this study, we compared the effects of single infections with *C. suis* (CS) and ETEC (EC), as well as co-infection (EC-CS) in weaned piglets. A comprehensive assessment of clinical signs, growth performance, intestinal pathology, barrier function, inflammatory responses, gut microbial community composition, and pathogen shedding demonstrated that *C. suis* and ETEC co-infection acts synergistically to enhance pathogenicity. The underlying mechanism involves interconnected pathophysiological processes, as summarized in Fig. 8.

As illustrated in Fig. 8, the synergistic mechanism is initiated by *C. suis*, which causes primary damage to the intestinal mucosa. The endogenous developmental stages of *C. suis* directly invade epithelial cells of the jejunum and ileum, resulting in villous atrophy and mucosal erosion [14]. The disruption of the intestinal environment leads to gut microbiota dysbiosis. Research by Shrestha et al. [49] confirmed that intestinal physical barrier damage caused by *C. suis* replication within enterocytes significantly reduces the alpha diversity of the piglet gut microbiota and alters its community structure; for instance, it leads to the enrichment of Proteobacteria, a hallmark of microbial dysbiosis. The microbial shifts observed in the co-infection model of this study likely originate from this primary enteric dysbiosis induced by *C. suis* infection [49, 50], which in turn creates a favorable niche for ETEC colonization. This plausibly explains why the fecal shedding of ETEC in the co-infected group was higher than that in the EC group during the early infection stages. Consistent with this, the dysbiosis in our model was characterized by reduced alpha diversity, a decrease in beneficial bacteria, and an increase in opportunistic pathogens including *Escherichia-Shigella* and *Clostridium* s.s. 1. The reduction of beneficial bacteria (e.g., *Lactobacillus*) diminishes the production of their anti-inflammatory metabolites. Concurrently, cell wall components (e.g., lipopolysaccharide) from overgrown opportunistic pathogens (e.g., Proteobacteria) are potent immunostimulants. Their persistent activation of immune cells (e.g., macrophages) triggers a substantial release of pro-inflammatory cytokines (e.g., TNF-α, IL-1β), thereby initiating and amplifying inflammatory responses. The co-infection group in this study exhibited the most significant upregulation of these pro-inflammatory cytokines, confirming an exacerbated inflammatory state. This robust inflammation can cause immunopathological damage, further compromise barrier function, and potentially influence disease progression by modulating host immune responses.

Regarding pathogen dynamics, co-infection not only increased early ETEC excretion but also enhanced the shedding of *C. suis* oocysts. This may be attributed to the pro-inflammatory environment induced by ETEC, potentially facilitating *C. suis* reproduction or oocyst formation. This finding is consistent with previous reports demonstrating that co-infections often lead to more severe outcomes. For instance, Mengel et al. [20] observed that co-infection with *Isospora suis* and *Clostridium perfringens* exacerbated clinical manifestations, increased mortality, and promoted the proliferation of *C. perfringens* in the jejunum. Similarly, Kirino et al. [51] documented a case of hemorrhagic enteritis in cattle resulting from co-infection with *Eimeria* and *C. perfringens*, with data indicating a positive correlation between oocyst counts and bacterial loads. In the context of viral–bacterial interactions, Lee et al. [15] provided direct evidence that co-infection with *C. perfringens* can enhance the replication of PEDV. Furthermore, Zhang et al. [52] reported that co-infection with PRRSV and PCV2 significantly elevated viral loads and pro-inflammatory cytokine levels, suggesting that the inflammatory milieu induced by co-infection may augment pathogen replication capacity. Collectively, this evidence supports the hypothesis that, in the co-infection scenario investigated in the present study, the inflammation triggered by ETEC may similarly facilitate the reproductive cycle of *C. suis*. Interestingly, by 9 dpi, the intestinal ETEC load was higher in the EC group than in the EC-CS group, suggesting that co-infection may lead to earlier activation of host mechanisms for clearing ETEC, or that the *C. suis*-induced alterations in the gut environment indirectly impair ETEC survival at later stages. The precise mechanisms underlying this observation merit further investigation.

Our analysis further revealed that different infection modes distinctly shaped the microbial community structures across the jejunum, ileum, and cecum. The characteristic microbial signatures associated with each group were as follows:

EC group (ETEC infection group): Marked by a significant expansion of Proteobacteria, particularly in the ileum (relative abundance 80.25%, a 17.36% increase over NC). This increase was driven primarily by the genus *Escherichia-Shigella*, a common pathogen positively correlated with diarrhea [1, 53, 54], reflecting successful colonization by Gram-negative pathogens, as supported by previous challenge studies showing that ETEC infection increases *Escherichia-Shigella* abundance [55]. A concomitant rise in *Helicobacter* was also observed.

CS group (*C. suis* single infection): Characterized by a notable increase in Actinobacteriota in the jejunum (6.93%, a 6.55% increase over NC), largely due to an abnormal rise in *Corynebacterium*, as observed in previous challenge studies where Actinobacteriota expansion was linked to diarrheal conditions [56]. A significant reduction in the beneficial genus *Lactobacillus* was observed in the jejunum (mean 68.78%, minimum 36.07%). This finding is consistent with previous studies wherein *Lactobacillus*, known for producing lactic acid and inhibiting pathogens, supports barrier function and mitigates inflammation; it is often enriched in healthy piglets but significantly diminished during diarrheal conditions [23, 54, 57]. In the present study, the reduction of this genus indicates that *C. suis* infection disrupts the probiotic barrier, potentially facilitating the proliferation of conditionally pathogenic bacteria. This pattern aligns with observations reported in prior research [49].

EC-CS group (co-infection): Exhibited a unique and dysbiotic microbiota, typified by an explosive proliferation of *Clostridium* s.s. 1 in the jejunum (13.95%, maximum 41.83%)—a reported biomarker for diarrhea risk [58]—alongside a compensatory increase in *Lactobacillus* in the ileum in some individuals (36.37%, reaching 71.27%). This paradoxical profile underscores the complex and potentially destabilizing influence of co-infection on microbial ecology, as seen in co-infection models involving ETEC and other pathogens [23, 59].

One potential limitation of this study is the choice to use weaned piglets as the model for co-infection with *C. suis* and ETEC. *Cystoisospora suis* is typically regarded as a primary pathogen in suckling piglets, and its pathogenicity may decline with increasing age [14]. A co-infection model in neonatal piglets could be more directly relevant under field conditions. This selection was driven primarily by our interest in the potential synergistic pathogenic mechanisms during the high-risk weaning period. Although previous studies have reported that *C. suis* infections are more frequent and severe in suckling piglets [14, 60], field surveys confirm that the parasite persists in weaned pig populations [61]. The weaning process involves significant stressors, including dietary changes and environmental transitions, which can lead to temporary immunosuppression and compromise intestinal barrier integrity [62, 63]. During experimental design, we hypothesized that even if the individual pathogenicity of *C. suis* is reduced in weaned piglets, the initial mucosal damage it causes might markedly increase intestinal susceptibility to ETEC strains associated with post-weaning diarrhea, potentially resulting in a more severe synergistic effect compared to single infections. However, it is important to acknowledge that studying *C. suis* infection dynamics in weaned piglets has inherent limitations. The severity of infection and patterns of oocyst excretion may differ from those in suckling piglets, which could influence the assessment of infection outcomes. In future work, we aim to explore co-infection with *C. suis* and other pathogens prior to weaning. We are also conducting large-scale epidemiological studies under conditions that better mimic actual production environments. This approach will help clarify the true economic impact of *C. suis* and ETEC co-infection during the post-weaning period.

Regarding the assessment of growth performance, the results indicated that although co-infection led to a significant reduction in body weight gain in the EC-CS group compared to the NC group at 7 weeks post-challenge, no statistically significant differences were observed following individual infection with either ETEC or *C. suis* alone. This lack of significant effect from the single-pathogen challenges could potentially be attributed to the limited sample size in this study, which may have constrained the statistical power to detect more subtle differences. Furthermore, the virulence and dosage of the challenged pathogens primarily induced short-term diarrheal symptoms, and compensatory growth during the recovery phase may have partially mitigated the impact of single infections on weight gain.

Considerable interindividual variability was observed in gut microbiota and cytokine profiles, a common limitation in studies using outbred animal models. This variability stems from inherent differences in baseline immunity and microbiota, the dynamic nature of host–pathogen interactions, and minor technical variations. Nevertheless, statistically significant trends across key indicators—including mortality, clinical scores, histopathology, and specific microbial taxa—robustly support our central conclusion of synergistic pathogenicity in co-infection. Future studies with larger cohorts or increased sampling frequency could more precisely delineate these dynamics.

From a clinical perspective, our findings underscore the fact that the diagnosis and control of piglet diarrhea must consider mixed infections. The presence of *C. suis* significantly potentiates the pathogenicity of ETEC, and vice versa. Thus, comprehensive strategies are essential, including (1) the use of anticoccidial drugs for early prevention of *C. suis*, (2) implementation of rigorous pen hygiene and disinfection to reduce environmental pathogen load [6], (3) application of probiotics to support intestinal health and microbiota stability, and (4) development of combination vaccines targeting co-infections.

This study establishes a foundation for understanding the interactions between two critical enteric pathogens in piglets. Future research should delve deeper into the specific molecular and immunological mechanisms driving the synergy, including changes in adhesion factors, inflammatory signaling pathways, and immune cell populations, as well as the impact of infection timing on disease severity.

## Conclusions

This study provides the first systematic demonstration of a synergistic pathogenic effect between *C. suis* and ETEC in weaned piglets. We confirmed that co-infection resulted in significantly more severe clinical symptoms and intestinal damage than single infections. The pathophysiological basis for this synergy involves a vicious cycle: *C. suis* acts as the initiator by disrupting the intestinal epithelial barrier, which is subsequently exploited by ETEC for enhanced colonization. The resulting enterotoxin secretion and robust inflammatory response further compromise barrier function and drive microbial dysbiosis, characterized by a decline in beneficial bacteria and proliferation of potential pathogens. This dysbiotic state reciprocally impairs barrier integrity, creating a self-amplifying loop of damage. Additionally, co-infection altered the excretion dynamics of both pathogens, delaying and increasing the peak output of *C. suis* oocysts while enhancing early-stage ETEC shedding.

The findings highlight the critical need for integrated control strategies against *C. suis* and bacterial enteritis in swine production. Isolated interventions are likely inadequate for managing such complex co-infections. Future development of more effective measures should target key nodes within this vicious cycle, such as preserving intestinal barrier integrity, modulating host immune responses, and maintaining microbial homeostasis.

## Supplementary Information

Additional file 1.

## Data Availability

The datasets used and/or analyzed during the current study are available from the corresponding author upon reasonable request. Raw sequencing reads have been deposited in the National Microbiology Data Center database for archiving (https://nmdc.cn/resource/genomics/metagenome).
